# Just-in-Time Adaptive Intervention to Improve HIV Prevention and Substance Use in Youth Experiencing Homelessness (MY-RIDE): Protocol for a Randomized Controlled Trial

**DOI:** 10.2196/78006

**Published:** 2025-10-10

**Authors:** Diane Santa Maria, Nikhil Padhye, Michael Businelle, Natasha Slesnick, Stefani Ricondo, Marguerita Lightfoot

**Affiliations:** 1 Cizik School of Nursing The University of Texas Health Science Center at Houston Houston, TX United States; 2 TSET (Tobacco Settlement Endowment Trust) Health Promotion Research Center University of Oklahoma Health Sciences Center Oklahoma City, OK United States; 3 College of Education and Human Ecology The Ohio State University Columbus, OH United States; 4 Pediatrics Adolescents, and Sports Medicine Baylor College of Medicine Houston, TX United States; 5 Oregon Health & Science University-Portland State University School of Public Health Portland, OR United States

**Keywords:** youth, homelessness, HIV, prevention, just-in-time adaptive interventions, mHealth, ecological momentary assessments, drug use, intervention, risk, mobile phone, mobile health

## Abstract

**Background:**

Youth who are experiencing homelessness face a higher risk of HIV infection compared to their housed peers, and suicide and overdose remain the leading causes of death among homeless youth. Just-in-Time Adaptive Interventions (JITAIs) are gaining momentum for HIV prevention and substance use research. Yet, most interventions for homeless youth have not addressed modifiable real-time factors.

**Objective:**

This paper describes the development and implementation of a randomized attention-controlled trial to assess the efficacy of motivating youth to reduce infections, disconnections, and emotional dysregulation (MY-RIDE), a JITAI to improve HIV prevention and substance use in homeless youth.

**Methods:**

This study will enroll 320 homeless youth aged 18-25 years. The intervention was co-designed with homeless youth using the Information-Motivation-Behavioral Skills Model and consists of an individual nurse-led session about HIV prevention and 3 months of a JITAI with personalized messaging delivered by phone in real time in response to one’s current level of risk. Participants also had access to an on-demand nurse helpline through the app.

**Results:**

Institutional review board approval was obtained in the summer of 2024. Recruitment began in the fall of 2024 at shelters, drop-in centers, and other organizations that serve homeless youth. Participants complete a baseline survey and HIV/sexually transmitted infection (STI) testing and are provided with a smartphone with the intervention app. Follow-up surveys and HIV/STI testing are conducted at immediate, 3-, 6-, and 12-month time points post intervention to assess uptake of HIV prevention strategies and substance use reduction. A total of 192 are enrolled to date.

**Conclusions:**

The results of this study will determine whether MY-RIDE increases HIV prevention strategies and decreases substance use when compared to homeless youth in the attention control group. We will also evaluate if MY-RIDE impacts protective factors such as willingness to take pre-exposure prophylaxis medication and use of mental health and substance use services, and antecedents of risk such as stress, substance use urge, and substance use.

**Trial Registration:**

Clinicaltrials.gov NCT06074354; https://clinicaltrials.gov/study/NCT06074354

**International Registered Report Identifier (IRRID):**

DERR1-10.2196/78006

## Introduction

There are an estimated 2.4 million people aged 18 to 25 years who experience homelessness in the United States [1]. The highest HIV risk groups—young men who have sex with men, youth of color, heterosexual Black young women, young adults who inject drugs, youth who engage in trade sex or are sexually exploited, and transgender young women who have sex with men all disproportionately experience homelessness [1,2]. While surviving the dangers of the streets and meeting basic needs, youth experiencing homelessness face enormous difficulties in maintaining their health and well-being [3,4]. Questions remain related to how to best address the persistent HIV disparities among homeless youth. Interventions that address substance use, mental health, and housing while also addressing multiple nonmedical drivers of health that marginalize homeless youth are critically needed [5].

Homeless youth are 6-12 times more likely to become HIV positive than their housed peers [6], with prevalence rates as high as 16% [7]. Yet, homeless youth lack adequate access to HIV prevention, including highly effective pre- and postexposure prophylaxis medications (ie, pre-exposure prophylaxis medication [PrEP] and postexposure prophylaxis medication [nPEP]) [8]. Despite the high proportion of homeless youth found to be eligible for PrEP (84%) in 1 large study, only 29% knew what PrEP was, and very few (14%) reported that they were actively trying to prevent HIV [9].

Substance use and stress increase the risk for HIV [10,11]. High stress predicts condomless sex, inconsistent condom use, more sexual partners, and substance use [12-18], with substance use rates among homeless youth being twice that of housed youth [3,19]. Nearly one-third of homeless youth also meet the criteria for co-occurring substance use and mental disorder diagnoses [20-22], and homeless youth often fail to recognize when they need treatment [23]. Barriers such as where and how to access services, costs, wait times, discrimination, and stigma associated with being homeless, having a mental illness, or using substances perpetuate chronic undertreatment [24-27]. The most common barriers to accessing care include lack of transportation, lack of health insurance, and health care costs [28]. Therefore, HIV prevention efforts must simultaneously address substance use and mental health needs and be readily accessible to be effective [29-32].

Homeless youth are disconnected from health care [33], are highly transient, and geographically mobile, making it difficult to engage in traditional brick-and-mortar care models. Only about half of street-dwelling youth and one-third of sheltered youth use health care regularly [34]. Additionally, homeless youth who use substances or have mental health issues experience compounding barriers to accessing mental health care [16] and HIV prevention measures, such as PrEP [35], with only one-third who need care accessing it [24,36]. While homeless youth continue to be underserved and understudied, prevention interventions specifically targeting homeless youth have achieved improvements in substance and alcohol use [37] and sexual health outcomes when addressing substance-using youth [38]. Interventions that address substance use, mental health, and housing while also addressing multiple nonmedical drivers of health that marginalize homeless youth are critically needed [5].

Ecological momentary assessments’ (EMAs) methodologies are also gaining momentum for HIV prevention and treatment research [39]. Using real-time, personalized motivational and prevention messages may provide more timely delivery of interventions. Yet, few HIV prevention mHealth (mobile health) interventions for homeless youth have been developed or rigorously tested [40]. Building on these mHealth efforts, this intervention uses EMA, Just-in-Time Adaptive Intervention (JITAIs) delivery, and on-demand nurse-led sessions that address substance use and stress management as an innovative, scalable, and accessible way to serve highly transient homeless youth [41].

**Figure 1:**
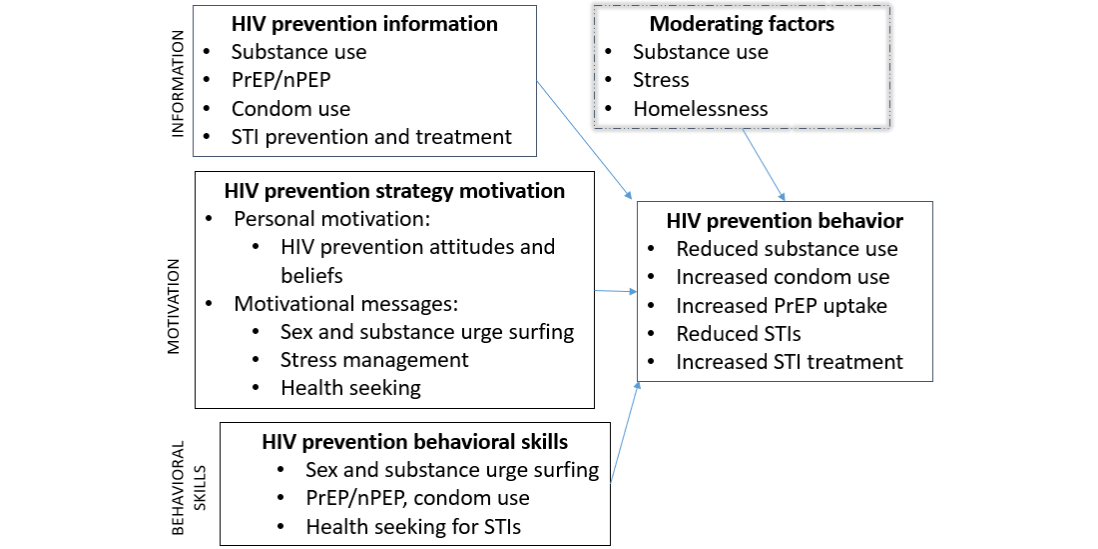
Information, motivation, and behavior guided MY-RIDE. MY-RIDE: motivating youth to reduce infections, disconnections, and emotional dysregulation; nPEP: postexposure prophylaxis medication; PrEP: pre-exposure prophylaxis medication; STI: sexually transmitted infection.

Motivating youth to reduce infections, disconnections, and emotional dysregulation (MY-RIDE) is an mHealth intervention that uses EMAs to predict imminent risk behaviors and delivers personalized messaging at the time of heightened risk. MY-RIDE addresses HIV risk and its antecedents in real time using messages developed with the Information-Motivation-Behavioral Skills (IMB) model and homeless youth [42]. This nurse-led model offers on-demand education sessions that use motivational interviewing (MI) [43-47] and shared decision-making (SDM) [48,49] approaches to promote behavior change and uptake of coping strategies by facilitating goal setting and increasing motivation and skills (Figure 1). The methods are integrated by using the IMB-informed messages in the app while the nurse delivers the HIV prevention content using MI and SDM strategies. Finally, homeless youth receive personalized messages delivered to their phone that address their current HIV risk factors as well as substance use, stress, and housing needs. Pilot data found that MY-RIDE decreased drug use, sexual urges, and stress among homeless youth [50].

Overall, the current study is guided by the IMB model [42,51]. IMB model posits that 3 primary factors influence behavior changes: information and knowledge about the behavior, in this case HIV risk behaviors; the individual’s motivation to perform the behavior; and the behavioral skills needed to modify the behavior [51]. Applying IMB, we hypothesize that homeless youth will reduce their risk for HIV when they (1) are provided with information on PrEP/nPEP, sexually transmitted infections (STIs), condom use, substance use, stress management, and health care services; (2) are motivated to change behavior by engaging in MI focused on HIV prevention and substance use reduction strategies with a nurse and receiving motivational messages in response to current stress symptoms; and (3) are provided with behavioral skills-building opportunities to reduce and manage stress, surf the urge to use substances or have sex, and increase health care seeking.

This randomized controlled trial will test the effects of MY-RIDE on HIV prevention in Harris County (Houston, Texas), 1 of the 48 Ending the HIV Epidemic counties that accounts for 50% of new HIV cases. The purpose of this trial is to determine whether MY-RIDE decreases substance use and increases HIV prevention strategies (condom use, PrEP uptake, and reduced STIs) compared to an attention control group of homeless youth (N=320; aged 18-25 years) immediately after the intervention and at 3-, 6-, and 12-months postintervention. An additional exploratory aim is to evaluate MY-RIDE effects on willingness to take PrEP, on stress levels, substance use urge, and use of mental health and substance use services when compared with attention control youth.

## Methods

### Study Design and Setting

This study uses a randomized 1:1 attention-controlled design to test the effects of MY-RIDE on substance use and HIV prevention among homeless youth. As there is some potential that EMAs or JITAIs can influence behavior, an attention-control design was chosen over a waitlist-control condition.

### Participants

Participants are being recruited from locations frequented by homeless youth, including the largest homeless youth drop-in centers and the largest homeless youth shelter in Houston, Texas. Both approximate the real-world service organization that would have the capacity to disseminate the intervention if found to be efficacious.

### Inclusion and Exclusion Criteria

The sample will include 320 homeless youth, aged 18-25 years, randomized 1:1 to either the intervention group or the control group. A random sequence generated in R (R Foundation) will be used for the simple randomization to ensure allocation concealment and minimize selection bias. Individuals at high risk for HIV will be included if they (1) have engaged in substance use (alcohol or illicit drugs) in the past 30 days, (2) are aged 18-25 years, (3) speak English, (4) are currently experiencing homelessness, (5) are engaged in sexual activity in the last 6 months or plan to in the next 30 days, and (6) are not planning to move out of the metro area during the 12-month study period. Current substance use is defined as having used any illicit drugs, drugs that are not prescribed, or alcohol in the past 30 days. Homelessness is defined as having slept on the streets, in a place not meant for habitation, a shelter, hotel or motel, or with someone where they cannot stay for more than 30 days (eg, couch surfing). This broader definition of homelessness aligns with the McKinney-Vento Homeless Assistance Act of 1987 and allows us to account for the transiency and instability of housing experienced by homeless youth, and increases the generalizability of this study’s findings. Homeless youth who have low literacy, based on the Rapid Estimate of Adult Literacy in Medicine-Short Form (scores <4), will be excluded due to the need to read independently at a 4th-grade level. We do not anticipate excluding many homeless youth based on this exclusion criterion. Only 1.2% of screened homeless youth from our prior study had low literacy [50].

### Data Collection

Data are being collected at baseline, immediately post intervention, and at 3, 6, and 12 months post intervention using REDCap (Research Electronic Data Capture; Vanderbilt University) on tablets or delivered through a web-based link that is password-protected and sent via text message on the study-issued phone. In-person survey takers are provided a quiet, private room (eg, at the shelter, drop-in center, and library) and most need about 30 minutes for completion of the survey. EMAs are collected 3 times a day for 12 weeks using the Insight App [52] and take about 1 minute to complete. The app is downloaded on this study’s issued smartphone to ensure all participants have functional devices on which to answer the EMAs and be contacted for study visits during the trial. To promote continuous engagement with the Insight app, EMA activity is monitored at 48-hour intervals, and participants are compensated based on percentage completion. Participants who do not complete an EMA in 48 hours are contacted on this study’s issued phone to verify if they are experiencing problems that can be addressed and to encourage EMA completion.

The presence of HIV antibodies, syphilis antibodies, and detection of chlamydia or *Neisseria gonorrhoeae* are being collected at baseline, immediately postintervention, and at 3, 6, and 12 months post intervention. In a private setting, participants complete a finger prick for rapid testing of HIV and syphilis and provide a urine sample for detection of chlamydia and gonorrhea. Tests were not diagnostic; all positive results are referred for confirmatory testing and treatment. Additionally, participants could provide study staff permission to access HIV/STI results from the on-site clinic when tested in the last 2 weeks to reduce testing burden.

### Instruments

We are using low-burden, empirically tested scales from our prior research with homeless youth [50,53]. The baseline survey includes demographics such as age, gender identity, race or ethnicity, sexual orientation, educational level, and employment. Historical factors are assessed to characterize the sample, such as foster care history, adverse childhood experiences [54], age at the first homelessness, and duration of current homelessness episode, all of which are mental health and substance use risk factors [55-58].

The primary outcome measure is substance use [59,60], measured with 2 items: “On many occasions (if any) have you used marijuana (weed/pot) in the last 30 days?” with responses ranging from 0 to 40+ and “select all drugs used in the past 30 days” with a range of drug options (not including marijuana). These items were separated based on feedback from the youth working group before the trial initiation. Due to the high use of marijuana in the population and the evolving laws around marijuana use, many youth did not consider it to be a drug. Descriptive statistics about the usage of marijuana and other types of drugs will enhance our understanding of trends in substance use in this population, while binary substance use (ie, use or nonuse of drugs, inclusive of marijuana) will be the primary outcome measure. One item in the daily EMAs “select drugs used yesterday” will be used to model changes in substance use over the intervention period compared to the intervention group.

Secondary outcomes are condom use, PrEP uptake, and STIs, and measurement was consistent with previous studies [50,53]. Condom use is assessed by two items: (1) “How often in the past 3 months did you use a condom during sexual intercourse?” with response options ranging from “never” to “every time” and (2) “The last time you had sexual intercourse, did you or your partner use a condom?” with response options of either “yes” or “no.” PrEP uptake is assessed by a single item: “Are you taking PrEP for HIV prevention?” with options “Yes, I take a pill for PrEP daily, Yes, I take a pill for PrEP sometimes, Yes, I get injectable HIV PrEP (a shot), No, I don’t take PrEP.” Presence of STIs is collected using biological specimens (blood and urine) by research staff or shelter clinic staff.

Willingness to take PrEP is measured in the follow-up surveys using an item consistent with previous studies [53,61], “How likely would you be to take PrEP if it were available for free?” with response options ranging from “Definitely would not take PrEP” to “Definitely would take PrEP”. The Perceived Stress Scale was also used, consisting of 10 items with response options from 0 (never) to 4 (very often), and can range from 0 to 40. Example item: “How often have you felt that you were unable to control the important things in your life?” Positively worded items are reverse-scored before scores are summed. This scale measures stress-related feelings over the last month, with higher scores indicating greater perceived stress [62,63]. Substance use urge is assessed in the EMAs with the item consistent with pilot study [50], “Right now, I am feeling a strong urge to: (Check all that apply): Steal, drink alcohol, use drugs, have sex, none of the above.” Usage of mental health and substance use services is measured using 2 items in the follow-up surveys: “In the last 3 months, have you used mental health (or substance use) services?” Response options are: “yes,” “no,” and “not sure.”

Measures from the PhenX Toolkit were used whenever possible to enhance data harmonization and data sharing efforts. EMA items measure real-time affect, stress, sexual risk behaviors, and urges (ie, right now), as well as real-time motivation measures. These Likert-type scale items have been successfully used with homeless youth and are based on our prior work and the work by Shiffman et al [50,64-67].

### Intervention Arm

An individual-level intervention design was chosen due to the heterogeneity of homeless youth and variability in real-time risk factors that impact HIV risk and substance use. The intervention, MY-RIDE, has three main components: (1) one nurse-led face-to-face session, (2) three months of EMA with personalized messaging delivered by phone in real time in response to one’s current risk, and (3) access to on-demand health care or PrEP navigation and referrals.

Nurses are ideal for leading the intervention sessions with homeless youth. Not only are nurses continuously ranked as the most trusted profession in the United States, an important consideration when working in a population with high levels of mistrust [68], but nurse-led interventions have the best potential for scalability and have been successful in improving HIV prevention strategies [69] among homeless youth, as they are trained to coordinate care across services through case management [53,70,71]. During the nurse-led face-to-face sessions, the nurse conducts an HIV risk assessment and assesses substance use, stress, and housing needs to guide the discussion about HIV prevention and goal attainment. The standard approach of the Screening, Brief Intervention, and Referral to Treatment tool is used for alcohol and drug use issues [72,73], with the nurse providing either a brief intervention or referral for substance use treatment. Sessions are held in a private setting such as a classroom, office space, library, or another empty room available at the community site.

The session allows for time to discuss HIV prevention strategies and establish a behavioral goal (ie, condom use, PrEP uptake, reduced substance use, sober sex, and reduced sexual partners). MY-RIDE builds on our previous prevention trial that supports the need for frequent, ongoing contact by providing ongoing prevention messages and booster sessions [50]. MY-RIDE participants are referred to local homeless youth-serving agencies to meet the complex, comorbid health or mental health, and social needs of homeless youth. MI and SDM strategies allow the nurse to tailor the discussion to the individual’s needs based on their current risks, chosen goal, and HIV prevention motivation level. The nurses are trained to follow the manualized MY-RIDE intervention session and complete a checklist of core activities to document delivery of Screening, Brief Intervention, and Referral to Treatment, PrEP eligibility, selection of prevention strategy, and referrals made after each intervention participant. Following the session, participants receive a study-issued cell phone with the app installed and general resources programmed for food, housing, and other local services for homeless youth.

**Figure 2:**
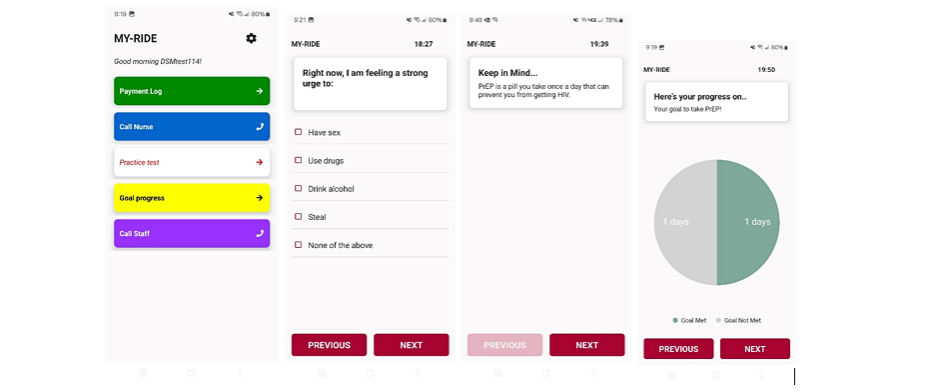
Insight app snapshots of the home screen, EMA item, intervention message, and behavioral feedback visual. EMA: ecological momentary assessment; MY-RIDE: motivating youth to reduce infections, disconnections, and emotional dysregulation; PrEP: pre-exposure prophylaxis medication.

The second component consists of 3 EMAs every day for 3 months with personalized messaging after each EMA using the Insight app [52]. Youth receive a targeted prevention message that responds to real-time risk levels at the moment the risk is detected. Real-time risk factors for substance use and HIV risk are based on previous studies and include stress, urge to steal, and urge or indication of sex, drug use, or alcohol use [50,64]. Decision rules for message delivery assign the appropriate message dose and content based on each participant’s current risk factors and relevant tailoring trait variables (eg, gender and sexual orientation) to deliver personalized, time-varying intervention content [65]. Over 900 intervention messages were co-developed with homeless youth and guided by the IMB model, and these were extensively beta tested and used successfully in the pilot [50]. In instances where multiple predictors are equally high, 1 message will be delivered with preference given to the strongest predictors (eg, real-time substance use). The justification for limiting to 1 message per EMA and the hierarchical ordering system based on prediction strength are grounded in the results from Projects Prevail and Smart-T [74,75], which indicated that receiving just 1 targeted message after each EMA reduced response fatigue. When low substance use and HIV risk are detected, a participant will receive more general messages reinforcing, for example, healthy stress management strategies, safety tips, or motivational quotes. Study staff reach out to youth weekly who have not responded to an EMA in 48 hours to assess for and address any challenges they may be experiencing (i.e., needing a phone charger). Additionally, the app automatically populates an interface that provides visual feedback about how one’s current behaviors align with their health goals, which can be accessed on their study-issued password-protected phone during the 3-month intervention period. This serves as an ongoing motivation to align or maintain one’s behavioral goals (Figure 2).

The third component of the intervention includes access to an on-demand nurse helpline. During the face-to-face session, the nurse demonstrates how the participant can reach this study’s nurse using a single “contact nurse” button on the app home page. Participants are advised to use this feature when they have questions about substance use, sexual health, or anything discussed in the face-to-face sessions. A registered nurse who is a part of this study’s team monitors this phone line and is prepared to answer questions and provide resources or local referrals regarding substance use, HIV/STI testing and treatment, PrEP or nPEP, and free condoms. Calls or texts are answered immediately or returned within 24 hours of a business day, and participants are directed to 911 if it is an emergency. Additionally, if a participant indicates that they need an STI test while completing a daily EMA, the nurse will automatically be notified via email and follow up with that participant to offer testing or navigate treatment seeking as needed.

### Control Arm

Participants assigned to the attention control arm will receive a 1-hour general health promotion session from a nurse, research coordinator, or research assistant and be issued a phone that will deploy the EMA on the same schedule as the intervention arm, with general health messages delivered after the EMA. These messages are unrelated to HIV risk, substance use, or its antecedents and address topics such as fruit and vegetable consumption, physical activity, water intake, and sleep hygiene. They will also receive usual care from the clinics, drop-in centers, and shelter recruitment sites. Usual care includes accessing social workers and case managers for immediate housing, food, and clothing needs and assistance in navigating the local continuum of care system to access social services. Specifically, youth are assessed at intake at the drop-in centers and shelters for mental health, substance use, and housing needs. This standard of care does not currently include HIV prevention education, personalized HIV prevention behavioral goal setting, or personalized HIV prevention messaging. Usual care has some evidence of reducing substance use in homeless youth, aged 14-20 years [76]. Attention control youth will also have access to the homeless youth’ resources programmed into the phone. While this is a low-risk study that will increase HIV prevention access and health care use, control youth will be monitored for adverse effects during this study and provided with the contact information for study staff, which will also be preprogrammed into this study’s phone.

### Sample and Power

The sample size (N=320) was determined from considerations of the MY-RIDE intervention effect on substance use (aim 1), STIs, PrEP use, and condom use (aims 2). For substance use, the effect size (odds ratio 0.61, 95% CI 0.39-0.97) was assumed from our previously published results that arose from a generalized linear mixed model framework [50]. Monte Carlo simulations implemented with the SIMR package [77] developed in the R programming language [78] yielded an estimated power of 0.96 for the intervention effect on substance use. Missing data were incorporated in the simulations by using a 50% response rate, from our pilot study, to EMA surveys over the 90-day intervention phase. Power for PrEP use was estimated similarly with a simulation that was based on the observed effect size in our ongoing National Institutes of Health–funded study that is testing a nurse case management intervention. The anticipated strong effect (odds ratio 10.59, 95% CI 2.44-50.90) was countered by the low prevalence of PrEP usage in youth, resulting in power=0.80 for the PrEP use outcome. The effect size (odds ratio 0.34, 95% CI 0.11-1.06) for STI was estimated from the aforementioned study, resulting in estimated power=0.81 for a 1-tailed test (and power=0.71 for a 2-tailed test). Lastly, the effect size (f=0.30) for condom use was estimated from a study involving youth of the same age group [79]. An estimated power=0.96 was determined using G*Power (version 3.1; Heinrich-Heine-Universität Düsseldorf) [80] for the intervention effect on condom use. In summary, estimated power for the primary and secondary outcomes ranged from 0.80 to 0.96 for a sample size of 320, randomized into 2 groups with 160 participants per group. All power calculations assumed α=.05 and included an allowance for 30% attrition, from our pilot study, from baseline to the first follow-up.

### Data Analysis

Baseline demographic characteristics (eg, age, gender identity, race or ethnicity, and sexual orientation) will be summarized with descriptive statistics in each arm. Random group allocation will make distributional differences of baseline variables unlikely. However, if any differences are found, the models testing intervention effects will include adjustment via propensity scores.

For aim 1, we determine whether MY-RIDE decreases substance use in homeless youth randomized to an attention control group (N=320; aged 18-25 years) at 0-, 3-, 6-, and 12-months postintervention. Bayesian mixed effects models will be used to test intervention effects on binary substance use. We hypothesize that at 3-, 6-, and 12-months follow-up, substance use will be lower in the intervention group than the attention control group. Models will use a logit link, and time will be treated as a discrete variable to allow nonlinear time dependence. Flat priors will be used for population-level parameters and Student *t* distribution for the SDs and cluster-level distributions, or random effects, with posterior distribution estimated with Hamiltonian sampling carried out using Stan [81]. Inference will be based on the statistical significance (*P*≤.05) of the interaction of time and intervention group. An adjustment term based on propensity scores will be included if demographic variables are unequally distributed in this study’s arms. The need for a random intercept and random dependence on time will be explored in these partially pooled models to allow variable trajectories between participants. Analysis for this study will be based on the intent-to-treat principle [82]. Multiple imputation will be used to safeguard against potential bias arising from the inclusion of only complete-case data. The *jomo* package for joint modeling will be used to specify a multilevel imputation model that is identical to the analysis model [83]. A similar approach will be used for analysis of substance use in the EMA data, with focus on longitudinal changes in daily reports of substance use over the intervention period. Time will range from 0 to 90 days for the EMA data on and it will be transformed, if necessary, for validating the assumption of linearity. Sensitivity analysis may be conducted if we have reason to believe that missing data are not ignorable [82].

For aim 2, we determine whether MY-RIDE increases HIV prevention strategies—condom use, PrEP uptake, reduced STIs—when compared with an attention control group of homeless youth. Intervention effects on condom use, PrEP uptake, and STI test results will be analyzed with Bayesian mixed effects models [84], following the method described in aim 1 above. We hypothesize that at 3-, 6-, and 12-months follow-up, the use of HIV prevention strategies will be higher in the intervention group than the attention control group. Condom use will be modeled as an ordinal measure (never to every time) and as a binary measure of condom use at the last sexual encounter. PrEP uptake will be modeled as a binary measure that combines the irregular use of PrEP with regular use because both are expected to be found in a small proportion of the data. STIs may be combined if the frequencies of individual STIs (eg, gonorrhea, chlamydia, and syphilis) are low, which will result in an any or none binary measure for STIs. Time will be treated as a discrete variable, and random intercepts will be included per participant. Statistical significance (α=.05) of the fixed effect corresponding to the interaction of time and treatment group will be used for inference of the intervention effect at each follow-up. The primary follow-up of interest is the one immediately following the intervention, while longer-term effects of the intervention will be judged from the follow-ups at 3, 6, and 12 months.

Analysis of the exploratory aim (evaluate MY-RIDE effects on willingness to take PrEP, on stress levels, substance use urge, and use of mental health and substance use services when compared with an attention control group of homeless youth) will also be conducted. Changes in time and by group will be modeled with a Bayesian mixed effects model similar to those described for aims 1 and 2. However, the emphasis will be on descriptive statistics and effect sizes in the models rather than on the statistical significance of the treatment effect. Models for EMA outcomes (mental health symptoms and substance use urge) will treat time as a continuous measure, while the models for exploratory outcomes (willingness to use PrEP, use of mental health, and substance use services) measured at the 3-, 6-, and 12-month follow-ups will treat time as a discrete measure. A simplified analysis of the changes from baseline in each exploratory outcome will be conducted to provide estimates that could be used for estimating the power in future studies.

### Ethical Considerations

Participant recruitment began after obtaining UTHealth institutional review board (IRB) approval (#HSC-SN-23-0360). Flyers describing this study are posted in the common areas of local shelters, drop-in centers, and clinics, as homeless youth often access services from multiple service providers and locations. Study information is disseminated during street outreach events hosted by partnering agencies. Local health care for the homeless providers, The Homeless Youth Network, and the Coalition for the Homeless were informed about this study, and flyers were posted at local clinics with permission, as well as housing first locations (where youth can access housing services). Project staff approach youth who receive services at drop-in centers and shelters such as Covenant House Texas to screen for eligibility. Additionally, participants can contact research staff after seeing flyers at other shelters, drop-in centers, or health care for the homeless clinics. The research staff is working with shelter staff to identify youth at the site each day who may be eligible. We are currently recruiting participants 3 days a week during regular business hours at the shelter and drop-in centers, and during street outreach. Research staff maintain a consistent presence at the agencies and approach young people who present to the agency. If the common room in the shelter or drop-in center is crowded, they will speak to the youth in a private office. The research staff explains this study and completes the informed consent process with interested youth. All prospective participants are assured that study participation will in no way affect their access to health and social services. Informed consent and contact information are obtained from participants.

Before this trial started, this study’s team met with the youth working group to review this study’s procedures, recruitment materials, survey questions, messages, and incentive amount and structure. Changes were made based on recommendations regarding the recruitment materials, wording, and response options for the survey questions, message wording, and incentive amount and timeline to enhance understanding, comfort, and fairness.

To ensure that potential participants do not feel coerced to enter or remain in this study, research staff recruiting participants will follow a standardized script to ensure that all ethical issues are reviewed and that this study’s procedures are followed with fidelity. All protocols were reviewed and approved by the IRB before implementation. It is made explicit in the informed consent document that participation in this study is voluntary, that it is unrelated to their access to health and social services, and that they can drop out of the research at any time for any reason. Participants will be verbally reminded at each follow-up that participation is voluntary and will not affect their ability to access any resources at the recruitment sites or at any other agency. Due to the potential for mistrust among homeless youth, participants will be reminded at each session and follow-up visit that the data collected in this study and the information shared will be confidential unless they share that they are thinking of hurting themselves or someone else. Participants will be reminded that all study staff are mandatory reporters and are required to report if they suspect that a participant is at risk of hurting themselves or others. Additionally, research staff reviewing the consent will be trained to probe for comprehension of the consent form and study procedures. A step-wise process previously used with this population [85] is used to ensure participants wait a minimum of 1 week between signing the consent form and officially enrolling in this study to provide additional time to think about being in this study. Agreement to be in this study is confirmed verbally before study enrollment.

Through our experience with homeless youth, the IRB, and the homeless youth working group, we have also set the incentive levels at a modest amount that is commensurate with the amount of time youth will spend in the research study and not deemed to be coercive. Incentives are provided as gift cards for survey and STI testing at baseline (US $40) and all follow-ups (US $40-$60) to approximate the amount of money one could earn in a similar amount of time. Additionally, participants are paid every month for EMA completion during the intervention period (3 months), ranging from US $25 to US $100, depending on the percentage of EMAs completed.

## Results

Institutional review board approval was obtained (#HSC-SN-23-0360) in the summer of 2024. Recruitment began in the fall of 2024 at shelters, drop-in centers, and other organizations that serve homeless youth. To date, there are 163 youth enrolled in this study. A total of 192 are enrolled to date.

## Discussion

### Anticipated Findings

This study will determine if MY-RIDE reduces HIV risk and substance use among a high-risk, hard-to-reach, vulnerable population of youth. The goal of this intervention is to reduce HIV risk by addressing risk behaviors, substance use, and stress among homeless youth through an innovative, theory-driven, mHealth intervention. MY-RIDE responds to current symptoms and imminent risk assessed using EMAs and delivers targeted, personalized information, motivational messages, and skill-building using a JITAI to increase symptom management and prevent related risk behaviors before they occur. We hypothesize that MY-RIDE will be more effective than attention control in reducing substance use and increasing HIV prevention strategies among young adults (aged 18-25 years) experiencing homelessness at 3, 6, and 12 months. While few interventions use mobile delivery of a JITAI to prevent HIV or substance use, the tenets of pairing an IMB-informed mobile app with a nurse-led model using MI and SDM are well-informed and supported by the literature.

### Prior Work

This intervention builds on our pilot study of MY-RIDE, which demonstrated reduced substance use, urge, and stress among homeless youth [50], though the current study was not powered to assess the intervention’s impact on HIV prevention strategies. Therefore, this study will be used to determine its efficacy for reducing HIV risk among homeless youth.

### Conclusions

MY-RIDE is grounded in a health equity lens that considers the social and structural determinants of success and nonmedical drivers of health, such as housing status and mental health, to find novel solutions to reach and engage youth who are transient and disconnected from services. By testing a novel and scalable mobile-based intervention, there is the potential to determine if MY-RIDE should be disseminated widely, offering health and social service providers a way to enhance HIV prevention among the youth that they serve.

## Abbreviations

EMA: ecological momentary assessment
IMB: Information-Motivation-Behavioral Skills
IRB: institutional review board
JITAI: Just-in-Time Adaptive Intervention
mHealth: mobile health
MI: motivational interviewing
MY-RIDE: motivating youth to reduce infections, disconnections, and emotional dysregulation
nPEP: postexposure prophylaxis medication
PrEP: pre-exposure prophylaxis medication
REDCap: Research Electronic Data Capture
SDM: shared decision-making
STI: sexually transmitted infection

## Data Availability

Data generated during this study will be available upon reasonable request to the corresponding author, following data sharing requirements per the National Institutes of Health and the University of Texas Health Science Center at Houston.
